# Living Newborn Back‐To‐Back With a Complete Hydatidiform Mole: A Rare Twin Pregnancy Outcome

**DOI:** 10.1155/crog/1897191

**Published:** 2026-04-29

**Authors:** Rime Abou Chakra, Wissam Arab, Nadine El Kassis, Malek Nassar, Karen El Ghoul, David Atallah

**Affiliations:** ^1^ Department of Obstetrics and Gynecology, Hotel-Dieu de France University Hospital, Saint-Joseph University, Beirut, Lebanon, usj.edu.lb

## Abstract

Twin pregnancy with complete hydatidiform mole (CHM) management is challenging and remains unguided up to this date. This report presents the case of a 20‐year‐old woman that was diagnosed with CHM coexisting with a healthy fetus at 18 week of gestation, following presentation with an episode of vaginal bleeding. After rigorous counseling about the significantly high risks of obstetrical complications and gestational trophoblastic neoplasia (GTN), the patient declined termination. Luckily, pregnancy was uncomplicated and successfully brought up to 34 weeks of gestation with serial follow‐ups, leading to an uncomplicated delivery via cesarean section. This condition presents unique diagnostic and management challenges, as clinicians must balance the potential for fetal survival against significant maternal risks.

## 1. Introduction

Twin pregnancy with a complete hydatidiform mole with coexisting fetus (CHMCF) is an extremely rare condition, occurring in approximately one in 22,000 to one in 100,000 pregnancies [1, 2]. Management is particularly challenging due to the high risk of complications, including intrauterine fetal demise (IUFD), early onset gestational hypertension and severe preeclampsia, antepartum hemorrhage, endocrine disturbances—most notably hyperthyroidism—and, most critically, the development of gestational trophoblastic neoplasia (GTN) [3, 4]. Additionally, these pregnancies have a high risk of miscarriage in the first trimester (40%) as well as an increased risk of premature birth (36%) and heavy vaginal bleeding requiring termination [5]. This report describes a case of CHMCF identified during the second trimester and highlights its distinctive ultrasound features, laboratory findings, and the intricate decision‐making involved in its management. Our report adds to the scarce literature on CHMCF and underscores the vital role of comprehensive patient counseling and careful postpartum monitoring in achieving optimal maternal outcomes.

## 2. Case Presentation

A 20‐year‐old primigravid woman with no significant medical or surgical history presented to the antenatal assessment unit following referral from her private gynecologist. The reason for referral was second trimester bleeding at 18 weeks along with a suspected anterior placenta praevia on imaging. The patient reported daily vaginal spotting for the preceding 5 days, soaking approximately one sanitary pad per day without passage of clots. On vaginal examination, brown‐red spotting was noted, with no evidence of active bleeding. Abdominal examination revealed a soft, nondistended abdomen with a second trimester gravid uterus.

Laboratory investigations showed mild anemia with a hemoglobin level of 10.6 g/dL. Serum β‐hCG was markedly elevated at 308,480 mIU/mL. Thyroid function tests revealed a suppressed TSH level at 0.13 mIU/mL with normal FT4 levels. The patient reported having suffered from hyperemesis gravidarum during the first trimester and said that her symptoms are still ongoing.

**Figure 1:**
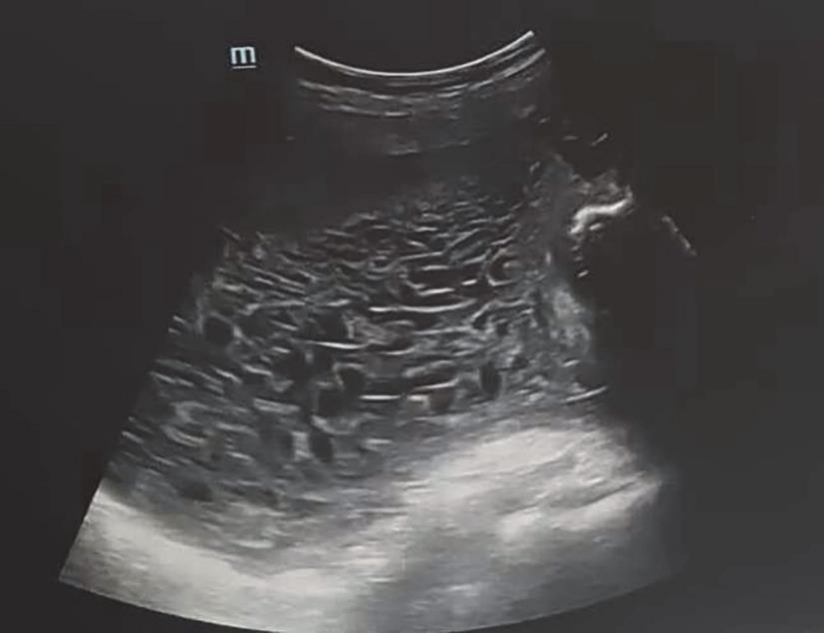
Snowstorm sign of of the molar part of the CHMCF (also known as honeycomb sign).

Obstetric ultrasound demonstrated a single live fetus corresponding to 18 weeks and 1 day of gestation, with an anterior placenta clear of the cervical os. Adjacent and inferior to the normal placenta, an enlarged heterogeneous placental mass with cystic and vacuolar changes was identified, measuring 13.1 cm × 4.2 cm and covering the cervix. These findings were consistent with a complete hydatidiform mole (CHM). The overall impression was a twin gestation with a live fetus and a coexisting CHM associated with anterior molar praevia (Figure 1). The fetus was morphologically normal and regularly grown for gestational age. The ultrasound findings at 18 weeks are summarized in Table 1.

**Table 1:** Obstetrical ultrasound scan biometry and morphology results.

Measured parameter	Value
BPD	39.5 mm
AC	124.5 mm
HC	147 mm
FL	26.3 mm
EFW	220–226 g
Cerebellum	16.6 mm, consistent with 16w6d GA
Lateral ventricle	5.44 mm

Abbreviations: AC, abdominal circumference; BPD, biparietal diameter; FL, femur length; GA, gestational age; HC, head circumference.

Given the significant maternal and fetal risks associated with a CHM coexisting with a live fetus, pregnancy termination was offered. However, the patient declined termination after counseling. She was therefore managed expectantly with close surveillance, home blood pressure monitoring, biweekly imaging along with serial bloods and β‐hCG measurements. The trend of β‐hCG levels during pregnancy and the postpartum period is presented in Table 2. The molar vesicular area remained stable in size and the patient was asymptomatic except for occasional spotting. Her follow‐up scans still showed a low lying molar area, which led to the recommendation of an early delivery.

**Table 2:** hCG level follow‐up (from 18 weeks up to 3 months postpartum).

18 weeks	268,195
20 weeks	249,685
22 weeks	258,573
24 weeks	164,889
26 weeks	158,835
1‐month postpartum	23.9
2‐months postpartum	17.8
3‐months postpartum	11.2

**Figure 2:**
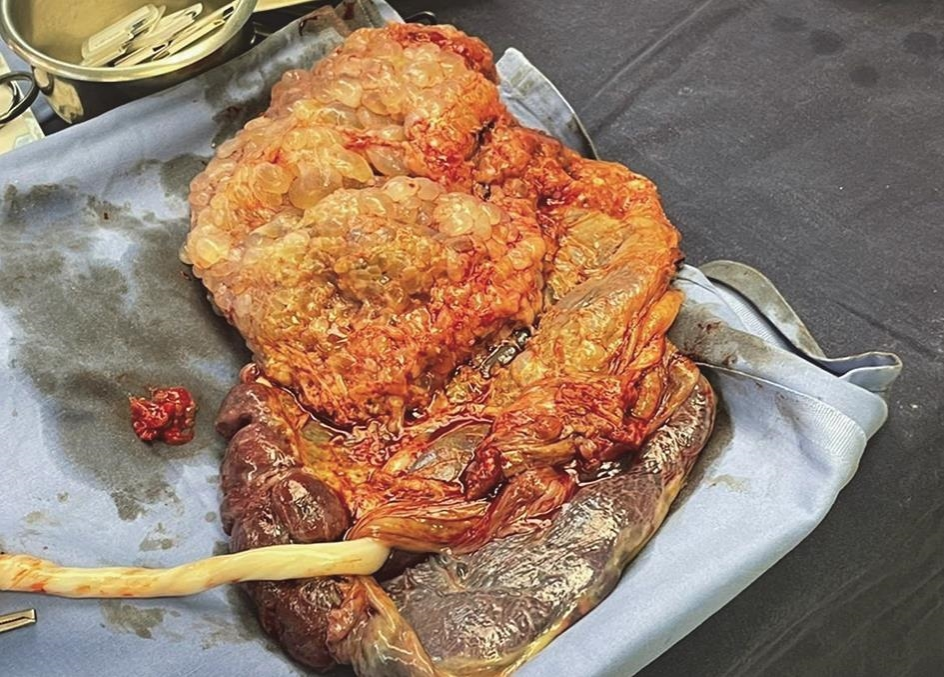
Fresh specimen in theatre showing the hydatidiform mole along with normal placenta. The CHM presents the typical “bunch of grapes” appearance and is directly attached to the normal placental tissue.

At 34 weeks’ gestation, a cesarean section was performed following administration of steroids for fetal lung maturation. A healthy neonate was delivered along with the CHM. The normal placenta along with the molar tissue were manually delivered during the procedure (Figure 2). Intraoperative blood loss was within normal limits.

The placenta was sent for histological examination. The specimen consisted of a discoid placenta measuring 20 cm × 16 cm × 3 cm and weighing 520 g, excluding the cord and membranes. The umbilical cord was centrally inserted, moderately twisted, measuring 18 cm in length and 1.2 cm in diameter, and contained three vascular channels on cross‐section. The membranes were translucent and covered with millimetric cysts originating from the complete mole. The specimen also included fragments of molar pregnancy. The detached molar tissue formed clusters measuring 18 cm × 16 cm × 4 cm, with a typical grape‐like appearance and cystic changes on sectioning. This molar tissue was partially adherent to the placenta. Microscopic examination revealed hydropic placental villi with a loose, edematous stromal core containing cistern formation. The villous surface was lined by hyperplastic trophoblastic tissue. These abnormal villi were seen in contact with, and adherent to, the membranes of the normal placenta. Short tandem repeat (STR) analysis demonstrated exclusive paternal alleles at all informative loci, with no detectable maternal contribution, confirming a diploid androgenetic origin consistent with a CHM.

Postpartum follow‐up demonstrated a progressive decline in β‐hCG levels, which returned to normal within 3 months after delivery (Table 2).

## 3. Discussion

Gestational trophoblastic disease (GTD) is a spectrum of uncommon conditions related to pregnancy and include the premalignant partial hydatidiform mole (PHM) and CHM, as well as the malignant invasive mole, among other GTNs [6]. In approximately 80% of the cases, a CHM presents with a 46, XX karyotype, arising from duplication of the haploid genome of a single sperm after fertilization of an ovum that has lost its maternal chromosomes during meiosis [7]. CHMCF generally results from a dizygotic conception in which one conceptus undergoes complete molar transformation. An apparent increase in the incidence of CHMCF has been reported in recent years, likely related to the growing use of assisted reproductive technologies [8].

The most common clinical manifestations of CHM, with or without a coexisting healthy fetus, include heavy vaginal bleeding, pelvic discomfort or pain, hyperemesis gravidarum, and an abnormally enlarged uterus. However, these symptoms are nonspecific and may also occur in a normal pregnancy. It is important to note that the clinical presentation of CHM has evolved with the widespread availability of ultrasonography and quantitative serum β‐hCG measurement, allowing earlier diagnosis and management [9]. The diagnosis of molar pregnancy is typically made by ultrasound between 12 and 14 weeks’ of gestation, with a reported detection rate of approximately 68% [10, 11]. Magnetic resonance imaging (MRI) may be considered when there is strong clinical suspicion but inconclusive ultrasound findings. On MRI, a hydatidiform mole appears as a heterogeneous mass with low signal intensity on T1‐weighted images and high signal intensity on T2‐weighted images [12]. In our patient, ultrasonography demonstrated heterogeneous cystic placental lesions with a characteristic “snowstorm” appearance, along with a single live fetus and a normal placenta. Histologic examination is always required in order to distinguish between PHM and CHM, and to rule out malignant transformation [6].

At diagnosis, β‐hCG levels are markedly elevated, typically peaking at the beginning of the second trimester [13]. Other markers, such as alpha‐fetoprotein (AFP), pregnancy‐associated plasma protein A (PAPP‐A), and pregnancy‐specific beta‐1 glycoprotein (SP1) may also be elevated. However, in CHMCF, AFP levels may remain normal [10]. In our patient, AFP and PAPP‐A were not measured considering that the clinical, laboratory, and imaging findings were highly suggestive of the diagnosis. Differential diagnosis between CHM and PHM is important as the risk of complication is higher in CHM with coexistent fetus compared to PHM with coexisting fetus. Generally, the USS findings in PHMCF are less prominent, and the increased placental echogenicity is more scattered due to focal hydropic degeneration [14]. HCG levels do not distinguish between the two entities and the accurate diagnosis is only obtained postnatally upon histology examination. Another differential diagnosis that should be considered is placental mesenchymal dysplasia (PMD). A rare entity, PMD is a nontrophoblastic placental vascular anomaly often associated with Beckwith–Wiedemann syndrome (BWS) or fetal growth abnormalities. Placental cystic changes seen on USS with PMD can mimic those of CHMCF; however, they are not seen separately from the placenta. In PMD, hCG and TSH levels are usually normal while AFP levels could be elevated [15]. Invasive diagnostic testing including chorionic villous sampling, and in rare circumstances placental biopsy, could be considered [16].

CHMCF is associated with several maternal and fetal complications, including preeclampsia, IUFD, hemorrhage, and venous thromboembolism. A meta‐analysis of 244 cases of twin pregnancies with CHMCF reported an overall incidence of antenatal maternal complications of approximately 80%. Preeclampsia occurred in 14.3% of pregnancies, and IUFD was reported in about 40% of ongoing pregnancies.

Regarding the risk of malignant transformation, current evidence suggests that the risk of GTN in coexisting molar and normal pregnancies compared with singleton molar pregnancies is increased from 15%–20% to 27%–46% [1, 3, 17]. Similarly, when compared to singleton molar pregnancies, CHMCF presents an increased risk of developing postmolar GTN and metastases that require multiple chemotherapy treatments [3]. The risk of GTN is similar between the patients who continue the pregnancy and those who terminate in the first trimester [18]. This highlights the importance of serial β‐hCG measurement, as levels correlate with disease burden and risk of complications. In our case, the progressive decline in β‐hCG levels during pregnancy was an additional factor supporting continuation of the pregnancy.

When CHM with a coexisting healthy fetus is diagnosed, elective termination of pregnancy has been recommended in many reports to prevent potentially life‐threatening maternal complications. In cases where the pregnancy is continued, the likelihood of a live birth ranges from 40% to 60% [6]. In a comprehensive review including 177 cases, the live‐birth rate was approximately 37% [19]. However, termination is not universally indicated if the fetus is healthy, and continuation of pregnancy may be considered with close monitoring of β‐hCG levels until normalization [20]. Whether termination or conservative management is pursued, it is preferred in our opinion to perform surgical evacuation or cesarean section to ensure evacuation of all molar tissue from the uterine cavity, and therefore, minimize the risk of retained molar tissue.

Regarding postpartum follow‐up, experts suggest to perform, in addition to serial hCG levels until negativation, a transvaginal USS before and early during a future pregnancy due to the increased risk of molar recurrence. A 6‐months contraception period is also advised.

In conclusion, CHMCF is a rare obstetric condition that poses significant diagnostic and management challenges. There is no clear consensus till this date about the best management approach, and available data from the literature is limited, mainly consisting of single case reports or collective reviews. Diagnosis requires a high index of clinical suspicion, characteristic ultrasound findings, and elevated serum markers. Although termination of pregnancy is usually recommended because of the relatively high risk of complications, continuation of pregnancy may be considered after thorough counseling and shared decision‐making. In such cases, close antenatal and postnatal surveillance is essential until normalization of serum β‐hCG levels.

## Funding

No funding was received for this manuscript.

## Consent

Written informed consent was obtained from the patient for publication of this case report and any accompanying images.

## Conflicts of Interest

The authors declare no conflicts of interest.

## Data Availability

Further data regarding the patient’s laboratory findings are available upon request.
